# Characterization of wound-induced electrical signals and reactive oxygen species in chickpea *(Cicer arietinum)*

**DOI:** 10.1080/15592324.2025.2567930

**Published:** 2025-10-30

**Authors:** Shweta Deshpande, Shivani Pawar, Archana Kumari

**Affiliations:** a Department of Plant Biotechnology, Gujarat Biotechnology University (GBU), Near Gujarat International Finance Tec (GIFT)-City, Gandhinagar, Gujarat, India; b Plant Molecular Biology Unit, Division of Biochemical Sciences, CSIR-National Chemical Laboratory, Pune, Maharashtra, India; c Academy of Scientific and Innovative Research (AcSIR), Ghaziabad, Uttar Pradesh, India

**Keywords:** Electrical signal, wound, ROS, chickpea, plant defense

## Abstract

Mechanical damage to plants triggers both localized and systemic responses that activate plant defense mechanisms. Early signaling events include calcium (Ca^2+^) flux, reactive oxygen species (ROS), and electrical alterations. These signals coordinate downstream defense pathways, enabling plant acclimation to biotic stress. Electrical signaling following wounding/herbivory has been extensively studied in *Arabidopsis*; however, its dynamics in crop plants such as chickpea (*Cicer arietinum*) are not well understood. The pattern of the SWP in chickpea was similar to that in *Arabidopsis* but with a longer repolarization phase and was detectable only within the leaflets. The signals generated by damaging the leaflet were more pronounced, propagated bidirectionally and varied between herbivore-susceptible and tolerant chickpea varieties. The SWP duration is correlated with increased expression of *AOS* and *OPR3* transcripts, which are markers of the stress hormone JA. Additionally, ROS production in wounded chickpea leaflets is associated with increased expression of ROS-generating genes. The use of DPI, an inhibitor of NADPH oxidase, which is responsible for ROS production, inhibited SWP, suggesting the crucial role of ROS in wound-induced SWP. This study provides insight into the interplay between wound-induced electrical signaling and ROS production in chickpea and proposes the measurement of electrical signals as a rapid, noninvasive approach for screening crop cultivars for pest susceptibility and tolerance.

## ARTICLE HIGHLIGHTS

1. Electrical signaling in chickpea follows the SWP pattern in response to wounding.
2. SWPs propagate bidirectionally with amplitude and propagation dependent on wound intensity.
3. SWP duration varies between herbivore-susceptible and -tolerant chickpea varieties.
4. Wounding triggers localized ROS accumulation in chickpea leaflets.
5. DPI attenuated SWP duration, highlighting the interdependence of ROS and electrical signaling.

## 1. Introduction

Injury to a plant triggers a cascade of changes in its cells, affecting their mechanical, chemical, and electrical properties.^1^ Mechanical changes primarily involve disruptions in cell wall integrity localized at the injury site.^2^ Chemical changes include Ca^2+^ ion flux,^3^^,^^4^ phytohormone modulation,^5–7^ amino acids,^8^^,^^9^ and the accumulation of reactive oxygen species (ROS),^10^^,^^11^ which collectively contribute to the plant’s defense mechanisms. Electrical activities in plants correlate with transient shifts in the plasma membrane potential, driven by ion movements such as Ca^2+^ influx and Cl^−^, K^+^, and H^+^ efflux.^12^ These signals constitute early responses to stress and are generated locally but are capable of propagating over long distances to initiate defense mechanisms in systemic tissues. Various models have been proposed to explain how mechanical, chemical, and electrical signals are integrated to trigger downstream defense pathways. One such model, the squeeze cell hypothesis, suggests that mechanical damage to cells disrupts the xylem water column, producing axial pressure changes that spread radially to neighboring vessels. “Squeezing” also triggers the activation of mechanosensitive ion channels (MSLs), which trigger membrane depolarization and Ca^2+^ influx. This further activates NADPH oxidase to produce ROS bursts. All these signals work in coordination and contribute to rapid signaling and systemic responses.^13^^,^^14^ There are three primary types of electrical signals in plants: action potential (AP), slow wave potential (SWP) or variation potential (VP), and system potential (SP).^15^^,^^16^ APs are typically triggered by touch and other nondamaging stimuli, representing a rapid response mechanism within plants. In contrast, SPs are associated with hyperpolarization and are induced by chemical treatments at wounds or herbivory.^17^ Leaf-chewing insects are known to generate SWPs in plants.^18^^,^^19^ Importantly, electrical signaling regulates diverse physiological processes, including stress hormone biosynthesis (abscisic acid, jasmonic acid), photosynthesis, respiration, phloem mass flow, and adenosine triphosphate (ATP) accumulation. ^20^^,^^21^

In legumes, SWPs or VPs also play certain physiological roles. For instance, treatment of pea protoplasts with the H⁺-ATPase inhibitor orthovanadate reduced the VP amplitude and photosynthetic activity and increased respiration, while the activator fusicoccin reversed these effects.^22^ In pea, VPs generated by heat reduced CO_2_ assimilation and transiently inactivated the dark reaction of photosynthesis in the affected/systemic leaf. These changes included lumen and stroma acidification, reduced PSII (Photsystem-II) electron flow, enhanced nonphotochemical quenching (NPQ), disrupted PSI electron transport and increased cyclic electron flow around PSI (Photosystem-I). Collectively, these responses contribute to photoprotection during systemic stress signaling.^23–27^ Similarly, heat-induced VPs increased respiration in *Vicia faba,*^28^ whereas wound-induced VPs in *Pisum sativum* stimulated polysome formation and inhibited protein synthesis.^12^^,^^29^^,^^30^ The SWP in *Arabidopsis* involves rapid (<2 seconds) and substantial (>50 mV) membrane depolarization, followed by a relatively longer (>2 minutes) recovery phase known as repolarization.^31^ The importance of glutamate-like receptors (GLRs), particularly GLR 3.3 and GLR 3.6, in regulating SWP in *Arabidopsis* was previously emphasized.^19^ Mutants lacking both *glr3.3* and *glr3.6* presented impaired Ca^2+^ transduction and shorter SWP durations,^32^^,^^33^ underscoring the critical interdependence of early signaling components. The coordinated effect of these components is essential for the activation of defense responses, with the absence of any single factor potentially disrupting the entire downstream signaling cascade. To measure the electrical activities noninvasively, surface electrodes are commonly employed. These electrodes capture the depolarization and repolarization events, which are quantified in terms of amplitude and duration, respectively.^18^ Wound-induced signaling is a critical aspect of the plant defense mechanism, enabling the rapid transmission of injury-related information to distal tissues. In *Arabidopsis*, injury triggers a robust SWP that propagates to the distal, parastichous leaves following the "n + 5 rule", where a leaf is vascularly connected to the fifth leaf from its position.^22^ *Arabidopsis* autoinhibitory H^+^-ATPase 1 (AHA1) has been identified as a key regulator that modulates SWP and the activation of the wound-induced jasmonate pathway. Compared with the wild type (WT), the *aha1−7* mutant presents a prolonged repolarization phase coupled with elevated levels of jasmonic acid (JA) and JA-Ile, as well as increased expression of jasmonate-zim domain protein 10 (*JAZ10*). This prolonged repolarization phase is associated with enhanced defense responses, indicating a crucial link between electrical signals and defense activation.^34^ In tomato (*Solanum lycopersicum*), both herbivory and mechanical wounding initiate electrical signals. Notably, wounding the petiole has a more pronounced effect on electrical signals than does wounding the leaf. The generation of SWP in tomato is correlated with the upregulation of *JAZ10* transcripts and increased levels of JA and JA-Ile,^35^ underscoring a conserved mechanism of the wound response across different plant species. Despite these advances in understanding electrical signaling, such mechanisms remain undocumented in legumes. We utilized chickpea as a model system to investigate wound-activated electrical signaling. Chickpea, with its typical uni-imparipinnate leaf arrangement,^36^ presents an opportunity to investigate potentially unique characteristics of wound-induced electrical signals compared with those of *Arabidopsis*. Similarly, ROS are important signaling molecules involved in coordinating systemic signals to different parts of plants.^37^ Mechanical injury to lima beans (*Phaseolus lunatus)* led to the accumulation of H_2_O_2_, mainly in the wounded region. Hydrogen peroxide (H_2_O_2_) production also triggered the upregulation of cytosolic Ca^2+^ and caused membrane depolarization in lima beans.^38^ The needles of the Pine plant *(Pinus sylvestris)* locally accumulated H_2_O_2_ in response to the eggs laid by the herbivorous sawfly *Diprion pini*.^39^ Gall midge infestation in wheat and rice plants increased the abundance of several ROS-producing genes and decreased the levels of many ROS-scavenging genes.^40^ All these studies highlight the importance of ROS accumulation for plant defense in the context of wounding or plant‒herbivore interactions.

The present study investigated the nature and propagation of wound-induced electrical signals in chickpea and focused on differences in SWP dynamics between herbivore-susceptible and -tolerant chickpea varieties. We examined the effects of wounding on ROS production and the expression of ROS-related genes. We further established the interdependence of ROS and electrical signaling by using an NADPH oxidase inhibitor. Our study revealed that the SWP pattern was similar in both *Arabidopsis* and chickpea, including rapid depolarization and slow repolarization phases. However, there were notable differences in the propagation dynamics of the electrical signals between the two species. Specifically, in chickpea, the SWP often fails to extend to systemic leaves, with its progression to systemic tissues depending on the severity of the injury. Our experiments provide valuable insights into SWP dynamics in chickpea, highlighting distinct variations between herbivore-susceptible and -tolerant varieties. Consistent with earlier findings,^34^^,^^41^ wounding chickpea leaflets resulted in the upregulation of markers associated with the JA pathway. Additionally, we investigated the impact of wounding on ROS accumulation and explored whether ROS inhibition affects electrical signaling in chickpea. This study aims to elucidate wound-induced electrical signaling and its interaction with ROS signaling. These results underscore that these two signaling processes are interconnected and activate plant defense responses. By elucidating this relationship, this research advances the understanding of how plants respond to injuries and initiate defenses against further damage and potential threats.

## 2. Materials and methods

### 2.1. Plant growth

Chickpea seeds (*C. arietinum L*.) of Vijay accessions were obtained from Mahatma Phule Krishi Vidyapeeth (Rahuri, India) and used in all the experiments. The ICCV 96970 and ICCV 92944 varieties were obtained from ICRISAT, India. The seeds were individually sown in plastic pots (height, 11 cm; diameter, 8.5 cm) with soilrite (Keltech Energies). The seedlings were maintained under controlled greenhouse (GH) conditions (light intensity: 150 µmol m^−2^ s^−1^; temperature: 23°C ± 1°C; photoperiod: 16:8: light:day) for the mentioned growth period. For electrical signal measurements, plants were transferred to a growth room with comparable growth conditions 1 d prior to the experiment.

### 2.2. Insect rearing

*Spodoptera frugiperda* eggs were procured from the ICAR-National Bureau of Agricultural Insect Resources (NBAIR), Bangalore, India. The insect larvae were reared on an artificial diet,^42^ and the moths were provided with 20% sucrose solution. Insects were reared under controlled conditions of 25°C ± 1°C, photoperiod: 16:8: light:day.

### 2.3. Surface potential measurement

Eleven- to thirteen-day-old chickpea plants grown in a GH were used to measure the surface potential changes. To measure the electrical activities noninvasively, surface electrodes are commonly employed. Electrical signals produced by the plants were recorded using a PhytlSign device (PSR2 HI) from Vivent Sàrl (Crans-près-Céligny, Switzerland) with an input impedance of 200 MΩ and a frequency of 256 Hz. The electrical potential was measured with custom-made electrodes optimized from the method described method.^43^ These electrodes capture depolarization and repolarization events, which are quantified in terms of amplitude and duration, respectively.^19^ To ensure a stable connection, the electrodes were affixed to the rachis surface with a solution of 20 µl of 10 mM KCl and 0.5% agar (HiMedia, Kennett Square, PA, USA). During the experiments, two silver‒silver chloride electrodes were placed on the rachis of either the same leaf (local) or different leaves (local and systemic), depending on the experimental setup. Additionally, reference electrodes were placed in the soil. The electrodes were allowed to stabilize on the rachis for 10 minutes before three or five terminal leaflets (>50% of the leaflet area) were wounded with forceps. For the experiments depicted in Figure 2c, 2d and Figure 4a, the leaflets of the local leaf were excised 1 d before the experiment. For diphenyleneiodonium (DPI) treatment, plants were treated with either 50 µM DPI or 50 µM dimethyl sulfoxide (DMSO) 30 minutes before their effects on ROS-related genes and electrical signaling were investigated. For the treatment, approximately 1 cm of the rachis, after the leaflets were removed, was wrapped with tissue paper, and 20 µl of 50 µM DPI or 50 µM DMSO was applied to the tissue. After 30 minutes, the leaflet between the two electrodes (both DPI-treated and DMSO-treated) was wounded. The surface potential changes were visualized as graphs, with amplitude (extent of depolarization) and duration (time taken for 50% repolarization) recorded in millivolts (mV) and minutes (min), respectively, as described previously.^19^

### 2.4. Plant-insect assay

For the plant-insect assay, 2.5-week-old chickpea accessions, namely ICCV 96970 and ICCV 92944, were used. Four preweighed 2^nd^ instar *S. frugiperda* larvae were left on each plant and allowed to feed for 4 d. Three plants per accession were considered. On the 4^th^ day, insect weight gain was measured, and plant damage was assessed. The assay was conducted under controlled conditions.

### 2.5. RNA isolation and quantitative real-time PCR (qRT‒PCR) analysis

**Figure 1.**
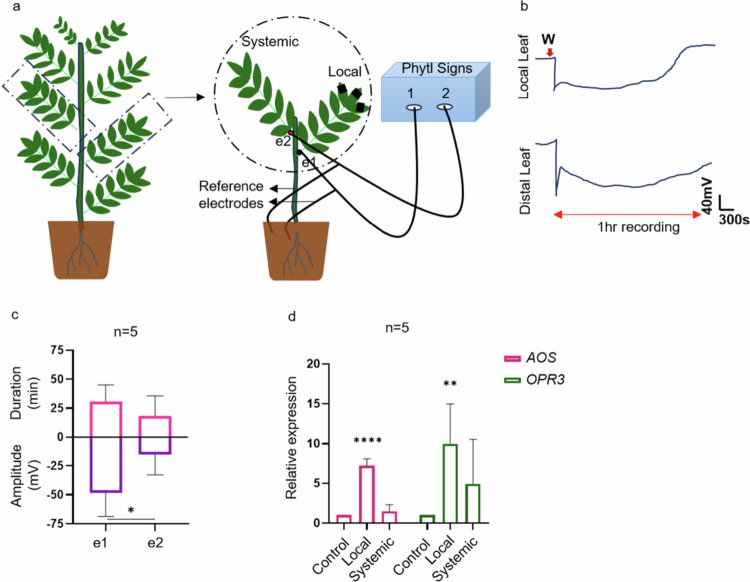
Wound-induced electrical signals in chickpea plants. The effects of wounding on electrical signals were measured in 11–12-d-old chickpea plants. (a) Experimental design for measuring electrical signals. The black and red dots represent the positions of electrodes e1 and e2, respectively. The black lines on the leaflet represent the area of wounding. Approximately 50% of the leaflet area is wounded using forceps. Recordings were measured for 1 hour. (b) Traces of electrical signals in local and distal leaves after wounding. (c) Amplitude and duration of SWP in local and distal leaves. (d) Relative expression of *Allene oxide synthase (AOS)* and *12-oxophytodienoate reductase-3 (OPR3)* in local and distal leaves after 1 h of wounding. The data presented are the means ± SD unpaired, two-tailed Students’ t-test. *P < 0.05.

Vijay plants were grown for two weeks under GH conditions. For experiments related to Figure 1d, three-terminal leaflets were wounded. For the experiments described in Figure 4c, the plants were treated with either DPI or DMSO (mock) for 30 minutes before wounding. Unwounded and/or untreated plants served as controls. For both experiments, leaf samples (local and/or systemic) were collected 1 hour after wounding and flash-frozen in liquid nitrogen. Total RNA was extracted using TRIzol (Invitrogen, Carlsbad, CA, USA) method. Complementary DNA (cDNA) was synthesized using the High-Capacity cDNA Reverse Transcription Kit (Applied Biosystems™). The PCRs were prepared with Takara TB Green® Premix Ex Taq™ (Tli RNase H Plus). qRT‒PCR was carried out on a 7500 Fast Real-Time PCR System (Applied Biosystems, Foster, CA, USA). The thermal cycling conditions included initial denaturation at 95°C for 30 seconds, followed by 40 cycles at 95°C for 5 seconds and 60°C for 30 seconds. This was followed by a melting curve analysis at 95°C for 15 seconds, 60°C for 15 seconds, and 95°C for 15 seconds. Gene expression fold changes were calculated using the 2^−(ΔΔCt)^ method,^44^ with the *elongation factor* (*EF-1α*) serving as the reference gene. The primer sequences are provided in Supplementary Table 2.

### 2.6. Confocal microscopy imaging

ROS imaging was performed on 2.5-week-old plants using a Stellaris V laser scanning microscope (Leica Microsystems CMS GmbH, Mannheim, Germany). For ROS detection, 20 µM dichlorodihydrofluorescein diacetate (DCFDA) dye (excitation/emission: 492 nm/517 nm, Invitrogen, Carlsbad, CA, USA) in 50 µM phosphate buffer (pH 7) containing 0.01% (v/v) Silwet L−77 was used. A total of 20 µl of dye was applied to each leaflet using a paint brush. The plants were initially treated with the dye for 30 minutes, followed by additional treatments or wounding with a gap of 20 minutes between subsequent treatment and wounding. After all the treatments/wound, the plants were incubated in the dark for another 30 minutes. The leaflets were subsequently visualized under a confocal microscope at 10× magnification.

### 2.7. Statistical analysis

All calculations and statistical analyses were performed using GraphPad Prism version 8.0.1.244 (GraphPad Software, San Diego, CA). Statistical significance was determined using Student’s two-tailed t-test, with a *P-*value of 0.05 regarded as statistically significant for all experiments. The data are expressed as the mean ± standard deviation (SD).

## 3. Results

### 3.1. Wound activated electrical signals in chickpea

We monitored the SWP in chickpea after wounding to determine whether chickpea exhibited a signal pattern similar to that of *Arabidopsis*. The experimental setup is illustrated in Figure 1a. Using the Vivent (PSR2) instrument, we observed typical SWP signals in *Arabidopsis* plants with comparable amplitudes and durations to those of the widely used WPI instrument^18^ for surface electrophysiology (Supplementary Figure 1). Furthermore, we employed a PSR2 instrument to record electrical signals in chickpea plants. Upon wounding the three terminal leaflets of chickpea (Vijay accession), the SWP was recorded at electrode e1. However, SWP was detected at electrode e2 (systemic/unwounded leaf) in only a few recordings (Figure 1b); Supplementary Table 1). The amplitude and duration of the SWP at local leaves were −48.2 mV and 30.8 minutes, whereas those at systemic leaves (e2) were −15.2 mV and 18.4 minutes, respectively (Figure 1c). Similar observations were recorded by Stahlberg and Cosgrove in 1992,^45^ where the SWP in response to stem excision in the pea plant failed to travel more than 10 mm from the injury site. On the other hand, burn stimuli in pea plants generate VPs that can travel to systemic leaves.^46^ The wound-induced electrical signal in chickpea shares characteristics with the SWP signal observed in *Arabidopsis*, although chickpea exhibits an extended repolarization duration compared with *Arabidopsis* (Supplementary Figure 1). To explore the connection between electrical signals and defense pathways, we analyzed the expression of *Allene oxide synthase* (*AOS)* and *12-oxophytodienoate reductase 3 (OPR3)* in both the local and systemic leaves of chickpea 1 hour after wounding. Transcripts of *AOS* and *OPR3* were upregulated in the local leaves (fold changes of 7.18 and 9.98, respectively). Upregulation was observed in systemic leaves (fold changes of 1.51 and 4.9, respectively) only where electrical signals were detected (Figure 1d; Supplementary Table 1).

### 3.2. Electrical signals travel distinctively through chickpea compound leaves

**Figure 2.**
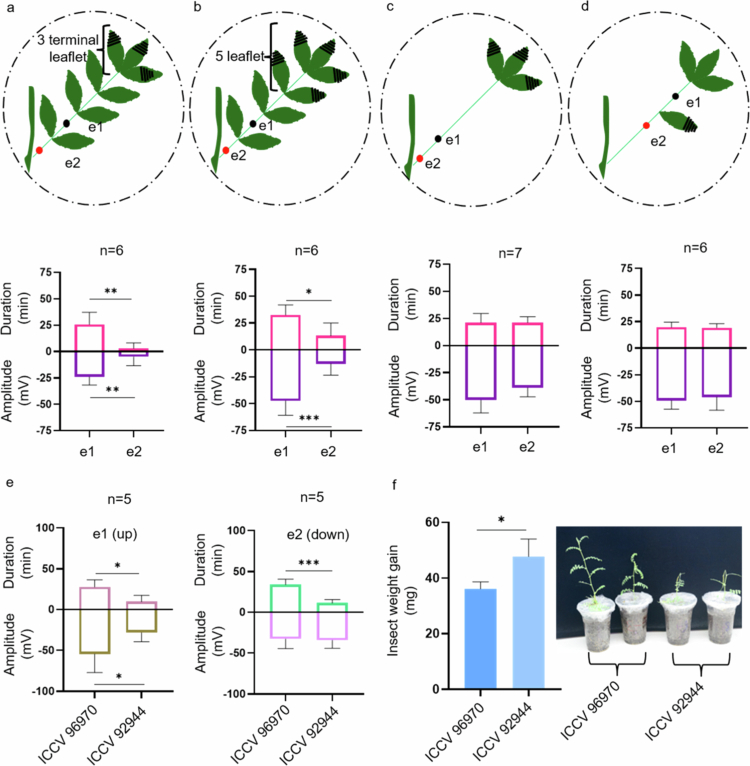
SWP propagation depends on leaf architecture and chickpea variety. The extent of SWP propagation in response to wounding was studied in 11–12-d-old chickpea plants. Experimental design for measuring the propagation of electrical signals and their corresponding quantified amplitude and duration at e1 and e2 in chickpea after (a) three-leaflet wounding, (b) five-leaflet wounding, (c) leaflet removal, (d) middle leaflet wounding (bidirectional movement of electrical signals). (e) Quantified amplitude and duration in susceptible ICCV 92944 and tolerant ICCV 96970 chickpea accessions at e1 and e2 after wounding the middle leaflet (as shown in setup-d. (f) Weight gain by 2nd instar *S. frugiperda* larvae after 4 d of feeding on susceptible ICCV 92944 and tolerant ICCV 96970 chickpea accessions and damage to the corresponding plants. The black and red dots represent the positions of electrodes e1 and e2, respectively. The black lines on the leaflet indicate the wounded area, which covers approximately 50% of the leaflet area using forceps. Recordings were measured for 40 minutes. The data presented are the means ± SD unpaired, two-tailed Students’ t-test. ^*^P < 0.05.

As signals could not be uniformly detected in systemic leaves, we hypothesized that signal propagation dynamics in chickpea differ from those in model plants such as *Arabidopsis* (Supplementary Figure 1). To investigate this, we measured how far signals can travel in chickpea when three terminal leaves are wounded. Initially, we tested 7−9-d-old plants with two to three expanded leaves and minimal stem distance between two consecutive leaves. The results were consistent with those of the previous experiment; signals were detected on the local leaf but not on the systemic leaf (Supplementary Figure 2). The chickpea plants have an alternate leaf arrangement, meaning that each leaf arises from a node positioned on the alternately opposite side. To avoid the possibility of vascular discontinuation with the nearest leaf due to its alternate-opposite position, we measured the signals on the systemic leaf on the same side as the local leaf. Nevertheless, no signal was detected in the systemic leaf (Supplementary Figure 3). Furthermore, we measured the signal on the same leaf with several leaflets. To measure the signal within the same leaf, we arranged the setup as shown in (Figure 2a), placing both electrodes on the same branch. Upon wounding three terminal leaflets of the chickpea, the SWP was detected at e1 (located two internodes away from the wound site) but not at e2 (3 internodes away). The amplitude and duration at e1 were −24 mV and 25.83 minutes, respectively (Figure 2a). When five leaflets were wounded (three terminal and an adjacent pair) (Figure 2b), the signals were detected at both electrodes, but the signals were significantly weaker at e2. The amplitude and duration at e1 were −47.3 mV and 32.5 minutes, respectively, and those at e2 were −12.8 mV and 13.16 minutes, respectively (Figure 2b). Distal signals in chickpea were inconsistently detected (Figure 2a) and were dependent on the intensity of wounding; more intense wounding resulted in longer signal propagation (Figure 2b). Chickpea leaves are typically unimparipinnate, meaning that 9−15 leaflets are attached to a common rachis.^36^ It is therefore possible that the components (mostly ions here) generating electrical signals diverge laterally to these leaflets rather than traveling along the rachis, dampening as they reach the far end. To investigate this hypothesis further, we removed all leaflets except the three terminals 1 d before the experiment (Figure 2c). When the three terminal leaflets were wounded, the signals were simultaneously detected at both electrodes. The amplitudes at e1 and e2 were −50.14 mV and −39 mV, respectively. The durations were similar (20.43 minutes at e1 and 21.14 minutes at e2) (Figure 2c). As shown in Figure 2c, in the absence of intermediary leaflets, the signals could be detected at both electrodes. In *Arabidopsis*, the SWP is known to propagate basipetally.^47^ To our knowledge, the bidirectionality of SWP in *Arabidopsis* has not been studied. For this purpose, an experiment was conducted with the setup depicted in Figure 2d. Prior to the experiment, the leaflets surrounding the electrodes were removed 1 d in advance. The leaflet positioned between the electrodes was mechanically wounded (50% of the leaflet area) using forceps and the electrical signal was measured. Remarkably, the resulting electrical signals were detected simultaneously, exhibiting similar amplitudes and durations (~50 mV and ~19 minutes) (Figure 2d). This observation confirms that electrical signals in chickpea plants are indeed bidirectional.

### 3.3. Potential of electrical signaling in identifying susceptible and tolerant varieties

We measured electrical signaling (similar to Figure 2d) in herbivory-susceptible (ICCV 92944) and herbivory-tolerant (ICCV 96970) chickpea accessions (unpublished data) after wounding. The amplitude and duration of SWP at e1 were significantly different between the two accessions, while at e2, only the duration of SWP was notably longer in ICCV 96970. The amplitudes at e1 for ICCV 96970 and ICCV 92944 were −54.6 and −28.36 mV, respectively. At electrode e1, the SWP duration was approximately 27 minutes for ICCV 96970, whereas it was 9 minutes for ICCV 92944. Similarly, at electrode e2, the SWP duration for ICCV 96970 was approximately 35 minutes, whereas it was 11 minutes for ICCV 92944 (Figure 2e). This observation aligns with data from the *Arabidopsis* mutant *aha1−7*, where a longer SWP duration (~3.5 minutes) relative to WT (~1 minute) was associated with increased plant resistance against *Spodoptera littoralis* and greater upregulation of *JAZ10*.^34^ Similarly, the extended SWP duration in the tolerant chickpea line ICCV 96970 may underlie its improved tolerance to *S. frugiperda*. This finding was supported by herbivory assays, where larval weight gain was higher on ICCV 92944 and significantly lower on ICCV 96970. This was also evident with more visible damage in the susceptible accession (Figure 2f).

### 3.4. Reactive oxygen species (ROS) accumulation in response to wounding

**Figure 3.**
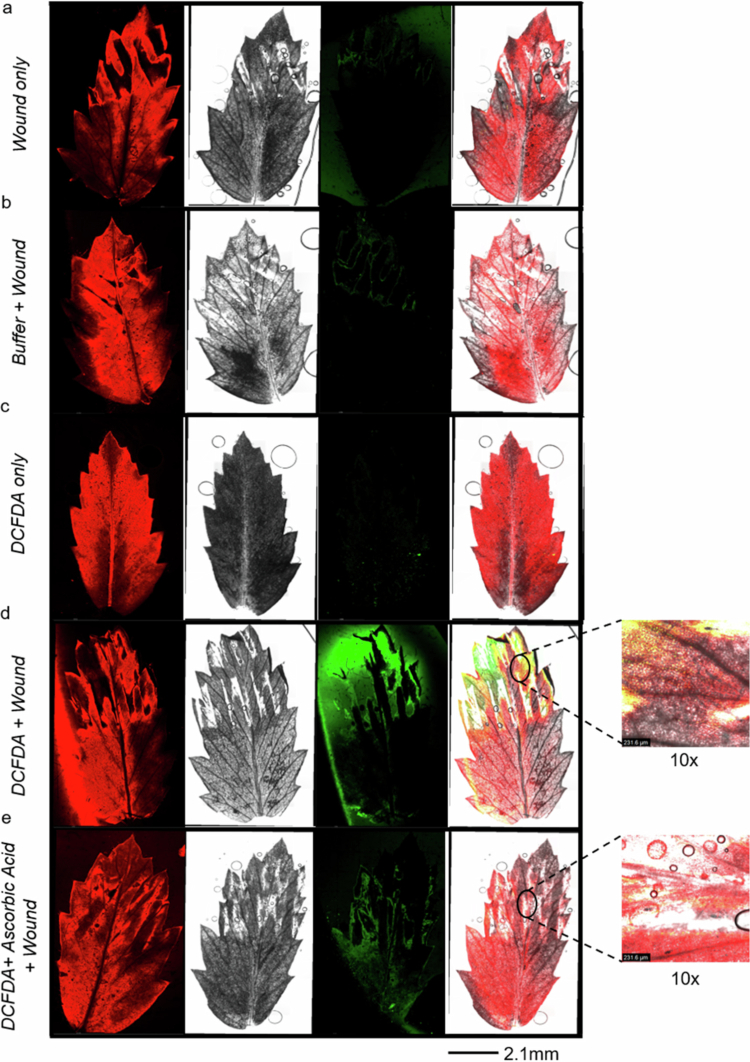
Wound-induced ROS accumulation in chickpea leaflets. Two-week-old chickpea plants were subjected to ROS detection with DCFDA dye. Leaflets from the 4th or 5th leaves were imaged at 10× magnification, and whole leaf images were reconstituted using spiral mode scanning with Leica software under various conditions: (a) Wound only, (b) Buffer + wound, (c) DCFDA only, (d) DCFDA + wound, and (e) DCFDA + ascorbic acid + wound. Whole leaf images were taken at a scale of 2.3 mm. Insets show 10× images of the wounded regions for conditions (d) and (e). The concentrations used were 20 µM DCFDA and 10 mM ascorbic acid.

Fluorescence microscopy imaging of the wounded chickpea leaflets treated with DCFDA dye revealed no fluorescence in the control samples; however, the wounded leaves presented green fluorescence localized to the wounded region (Figure 3a–d). The introduction of ascorbic acid prior to wounding resulted in quenching of green fluorescence, indicating that ascorbic acid efficiently scavenges ROS (Figure 3e). As a positive control for dye penetration and ROS detection, leaves treated with 1 mM H₂O₂ presented strong green fluorescence, whereas those treated with DCFDA alone did not produce a signal (Supplementary Figure 4). These results suggest that wounding induces stress-related accumulation of ROS in chickpea leaflets within 30 minutes, as evidenced by the oxidation of DCFDA, and that ROS accumulation is confined to the wounded area.

### 3.5. Effect of ROS inhibitor on wound-activated electrical signal

**Figure 4.**
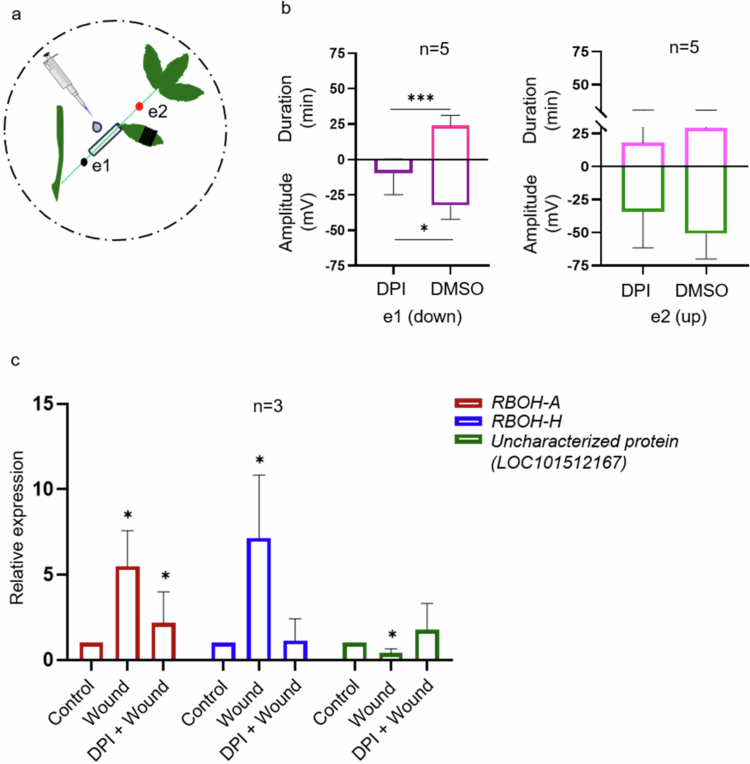
DPI attenuates SWP and affects the expression of ROS genes. The effects of the NADPH oxidase inhibitor DPI on SWP and ROS gene expression were assessed in 2-week-old plants. (a) Experimental design, (b) amplitude and duration after 30 minutes of DPI or mock (DMSO) treatment. The black and red dots represent the position electrodes e1 and e2, respectively. The black lines on the leaflet indicate the wounding area, with approximately 50% of the leaflet area wounded using forceps. Recordings were measured for 30 minutes. (c) Relative expression of *respiratory burst oxidase homolog A (RBOH-A), respiratory burst oxidase homolog H (RBOH-H)*and an uncharacterized protein (LOC101512167) in local leaves 1 hour after treatment. The data presented are the means ± SD unpaired, two-tailed Students’ t-test. *P < 0.05.

We treated the plants with DPI, a chemical inhibitor of NADPH oxidase,^48^ to investigate the effects on electrical signaling (Figure 4a). DPI treatment shortened the SWP amplitude at e1, and the repolarization duration was almost zero minutes at e1 compared with that of the DMSO-treated plants. The DPI treatment had no significant effect on the SWP at e2 (Figure 4b). These findings suggest that SWP was affected in the DPI-treated area but not in the opposite direction, suggesting that bidirectional movement is independent.

We also wanted to test the effect of wounding on the expression of ROS-related genes. For this purpose, genes were shortlisted based on RNA sequencing data of herbivore-susceptible and herbivore-tolerant varieties subjected to simulated herbivory (unpublished data). qRT‒PCR analysis revealed the upregulation of the following ROS-generating genes: the respiratory burst oxidase homologs *RBOH-A* and *RBOH-H*. The expression of the ROS-scavenging gene, an uncharacterized protein (LOC101512167) with homology with the *Arabidopsis* glutaredoxin gene *At5g39865*, was also significantly reduced in wounded leaves (Figure 4c). Since DPI treatment attenuated the SWP, we further wanted to test whether it affects the expression of the above ROS-related genes. The DPI + wound treatment led to significant but diminished expression of *RBOH-A* compared with that in only wounded plants. The treatment had a more profound effect on *RBOH-H,* where the expression was comparable to that of the control samples. On the other hand, the expression of an uncharacterized protein (LOC101512167) was similar to that of the control sample in the DPI + wound treatment, which was otherwise downregulated in the wound-only treatment (Figure 4c). These findings suggest that DPI inhibits the activity of ROS-generating genes, leading to decreased ROS accumulation in response to wounding, which subsequently attenuates electrical signaling. This study provides evidence linking ROS levels and electrical signaling in chickpea.

## 4. Discussion

Activating defense responses is a key strategy employed by plants to survive multiple stress factors in the environment. Some of the early signaling responses in response to wounding/herbivory include electrical signaling, Ca^2+^ fluxes, and ROS accumulation.^9^^,^^31^^,^^35^^,^^49^ These signals can travel long distances, thereby alerting the plant to a potential threat. The evidence suggests that membrane depolarizations can spread symplastically via plasmodesmata or along the plasma membrane of sieve elements.^30^^,^^50^ The SWPs travel through the primary veins of *Arabidopsis* at apparent velocities of 6–9 cm minute^–1^,^19^^,^^32^ whereas in pea epicotyl, SWPs travel 20−30 mm minute^–1^.^51^ While these responses and their implications for plant defense are well characterized in the model plant *Arabidopsis*,^4^^,^^11^^,^^34^ there is very limited knowledge about these signaling components in other crop plants. Here, we sought to characterize the responses of the legume chickpea. An SWP is characterized by a rapid membrane depolarization phase followed by a long repolarization phase.^31^ Our findings indicate that chickpea generates an electrical signal, specifically a slow wave potential (SWP), similar to that of pea^45^^,^^46^ and *Arabidopsis* but with shorter amplitude changes and longer repolarization times (Figure 1c, Supplementary Figure 1). Unlike the traditional SWP pattern, where a decrease in membrane potential occurs after 1−2 s of injury, depolarization in chickpea is rather immediate and occurs spontaneously in the local and distal parts of the leaf (Figure 1b). Electrophysiological studies in other legumes provide valuable context. In 1992, in pea, basal stem excision induced SWPs with 20–50 mV depolarization and ~15 minutes of repolarization,^45^ a pattern similar to our observations, though chickpea exhibited longer repolarization. In *Vicia faba*, apoplastic SPs were reported after wounding,^52^ while *Phaseolus vulgaris* (common bean) exhibited >50 µV depolarizations, which were restored within minutes.^53^ In lima bean (*Phaseolus lunatus*), herbivory by *Spodoptera littoralis* triggered strong depolarizations within 1.5 mm of bite sites, which propagated across the leaf. Mechanical wounding or single cut-initiated membrane potential drops and the application of insect oral secretions also resulted in clear depolarization responses.^54^ In chickpea, we observed that wounding triggered SWP production but with limited propagation, which was typically confined to local tissues. In common beans, depolarization was observed following herbivore feeding as well as after both repeated and single mechanical wounding events, with the response lasting for several hours before gradually returning to baseline levels. The application of *S. littoralis* regurgitation after mechanical treatment also led to clear depolarization responses.^55^

The immediate depolarization is a characteristic of AP. In cases such as wounding, the electrical signals are often a mix of AP and SWP and at times, they overlap, making them difficult to distinguish. The overlapping nature of AP and SWP has also been reported in excitable plants such as *Dionaea* and *Mimosa*.^56–58^ SWP propagation was also correlated with the upregulation of JA synthesis markers, *AOS* and *OPR3* (Figure 1d), in local leaves, suggesting that early signaling components activate downstream defense pathways.^17^^,^^34^ Systemic signals are crucial for priming defenses in undamaged tissues and organs. A study revealed that a damaged dandelion leaf can transmit electrical signals to an undamaged leaf of a neighboring dandelion in physical contact, highlighting plant networking and defense priming via electrical signal propagation.^59^ In the case of chickpea, the failure of the SWP to reach systemic tissues after three node intervals (Figure 2a) may be attributed to the uni-imparipinnate leaf architecture of chickpea.^36^ The signal appeared to be diluted among the lateral leaflets, and when the intervening leaflets were removed, the SWP signal could be detected even after three node intervals. Two possible explanations for this observation are as follows: (1) a relatively high volume of liquid in the xylem and (2) the complex leaf architecture of chickpea, where the SWP might dilute across the leaflets, hindering long-distance travel. By removing the leaflets, the SWP had no lateral divergence and thus travelled a longer distance more efficiently. These observations suggest that leaf architecture significantly influences signal propagation, highlighting a potential area for further research on the anatomical and physiological factors affecting electrical signaling in different plant species. Previous studies have consistently implicated pressure changes in the xylem and deformation of its vessel walls in SWP initiation.^18^^,^^51^^,^^60^^,^^61^ In the case of *Arabidopsis*, electrical signals travel basipetally from sites of damage to the primary vein and then disperse into leaves that share direct vascular connections with the damaged leaf.^47^ An intriguing aspect observed in chickpea is the bidirectional nature of the SWP (Figure 2d), potentially explained by the release of water pressure along the xylem in both directions when the middle leaflet is wounded (Figures 2d and 4b). This bidirectionality may suggest a unique adaptation in chickpea for coping with localized damage, ensuring comprehensive signal propagation throughout the plant. Further studies are needed to confirm this observation, possibly involving detailed analyses of xylem pressure dynamics and the physical properties of chickpea vascular tissues.

SWP propagation occurs in two steps: first, through the combined stimuli of hydraulic waves and chemical factors. The second step is the self-propagation step, which is achieved through changes in cytosolic calcium, ROS, and membrane depolarization.^14^^,^^50^^,^^57^^,^^62^^,^^63^ In plants, ROS play a significant role in mitigating abiotic and biotic stress responses, integrating stress signals, and activating downstream defense responses.^64–66^ Fluorescence imaging revealed that ROS accumulated in the wounded region of the leaflet in response to wounding (Figure 3d). The NADPH oxidase inhibitor DPI reduces the expression of two key ROS genes, *RBOH-A* and *RBOH-H* (Figure 4c). In addition to affecting ROS levels, DPI treatment also influenced the SWP in chickpea. The amplitude of the wave was significantly attenuated, and the repolarization duration was negligible under DPI treatment (Figure 4b). It has been reported in *Arabidopsis* that, when wounded, systemic changes in ROS, Ca^2+^, membrane potential, and hydraulic signals all require functioning GLR3.3 and GLR3.6.^37^ Here, through DPI experiments, we indirectly established a link between ROS and electrical signaling in chickpea, although molecular studies are needed to establish a concrete connection.

This study was extended to investigate the electrical signaling pattern across chickpea varieties susceptible and tolerant to herbivores. The electrical signaling pattern observed in susceptible and tolerant chickpea accessions aligns with findings in the *Arabidopsis* mutant *aha1−7*, where prolonged repolarization phases were associated with increased resistance. The differences in SWP duration between tolerant (ICCV 96970) and susceptible (ICCV 92944) accessions suggest that electrical signaling could serve as a potential tool for assessing plant resistance. While molecular markers such as restriction fragment length polymorphism (RFLP), random amplified polymorphic DNA (RAPD), amplified fragment length polymorphism (AFLP), simple sequence repeats (SSR), and single nucleotide polymorphisms (SNPs) have been used for cultivar identification, population genetics, quantitative trait locus (QTL) mapping, disease identification, and other applications,^67^^,^^68^ these methods are often time consuming, labor intensive, and costly. In contrast, electrical signaling offers a rapid, sensitive, and efficient alternative for high-throughput screening of crop cultivars for susceptibility and tolerance phenotypes. Furthermore, it enables early detection of resistance traits, which can be further utilized to elucidate the underlying mechanism of tolerance.

## 5. Conclusions

This study demonstrates the dynamics of electrical signaling in chickpea compared with *Arabidopsis*. The distinct responses observed in chickpea, such as the prolonged repolarization time and the unique bidirectional nature of SWP, suggest that different plant species have evolved specific mechanisms to optimize their defense responses. Furthermore, the interaction between electrical signaling and ROS signaling pathways highlights that these pathways are interconnected and synergistic in activating and amplifying plant defenses that help mitigate further damage and protect against potential threats. Future research should elucidate the molecular mechanisms behind the observed differences in electrical signaling between chickpea and *Arabidopsis*. This includes identifying key genes and proteins involved in signal generation and propagation and examining the impact of various environmental conditions. Additionally, studying the interplay between electrical signals, hormonal responses, and Ca^2+^ ions in chickpea will provide a comprehensive understanding of plant defense mechanisms. It would also be interesting to study electrical signaling in other plants with a unimparipinnate type of leaf arrangement. These insights can enhance our understanding of plant responses to environmental stressors, leading to improved agricultural productivity and sustainability.

## Supplementary Material

Supplementary material **Supplementary Table 1.** Wound-induced electrical signals (amplitude and duration) and fold changes in the expression of JA markers (AOS and OPR3) in local and distal leaves.

Supplementary material **Supplementary Table 2.** Details of the primers used for qRT‒PCR analysis.

Supplementary material **Supplementary Figure 1.** SWP pattern in Arabidopsis: SWP was measured in 6-week-old Col-0 lines of Arabidopsis using the PhytlSigns instrument. Leaf 3 was wounded, and signals were recorded in both wounded local leaf-3 and systemic leaf-8. (a) Schematic of the experimental setup for monitoring wound-induced SWP changes. (b) Amplitude and duration of SWP in local and systemic leaves. The data represent the means ± SD.
**Supplementary Figure 2.** Electrical signaling in seven to nine-day-old chickpea plants: The electrical signaling was measured in smaller plants with shorter internodal distances. The graph represents amplitude and duration of SWP observed in chickpea seedlings following the wounding of three-terminal leaflets. Data represented are means ± SD. Unpaired, two-tailed Students’ t-test *p<0.05.
**Supplementary Figure 3.** SWP fails to propagate to the same-side systemic leaf: (a) Experimental design to measure the propagation of electrical signals to the same-side systemic leaf after a three-leaflet wound. (b) The amplitude and duration of SWP were measured in both the local (wounded) and systemic (same side) leaf of chickpea. Black and red dots represent the position of electrodes e1 and e2 respectively. The black lines on the leaflet depict the wounded area, with approximately 50% of the leaflet surface wounded using forceps. Recordings were measured for 30 minutes. Data represented are means ± SD.
**Supplementary Figure 4.** Evaluation of DCFDA cell penetration: To control for dye penetration, leaflets were initially treated with 20 µM DCFDA followed by 1 mM H2O2 treatment, and subsequently imaged for ROS fluorescence (upper panel). Leaflets treated with DCFDA alone served as the negative control (lower panel).

## Data Availability

The data that support the findings of this study are available from the corresponding author upon reasonable request.
