# Supplementary material for: Characterization of wound-induced electrical signals and reactive oxygen species in chickpea (Cicer arietinum)
Source: Plant Signal Behav. 2025 Oct 30;20(1):2567930. doi: 10.1080/15592324.2025.2567930 (PMC12584604; doi:10.1080/15592324.2025.2567930)
Supplement: Supplementary material — Supplementary Figure 1. SWP pattern in Arabidopsis: SWP was measured in 6-week-old Col-0 lines of Arabidopsis using the PhytlSigns instrument. Leaf 3 was wounded, and signals were recorded in both wounded local leaf-3 and systemic leaf-8. (a) Schematic of the experimental setup for monitoring wound-induced SWP changes. (b) Amplitude and duration of SWP in local and systemic leaves. The data represent the means ± SD. Supplementary Figure 2. Electrical signaling in seven to nine-day-old chickpea plants: The electrical signaling was measured in smaller plants with shorter internodal distances. The graph represents amplitude and duration of SWP observed in chickpea seedlings following the wounding of three-terminal leaflets. Data represented are means ± SD. Unpaired, two-tailed Students’ t-test *p<0.05. Supplementary Figure 3. SWP fails to propagate to the same-side systemic leaf: (a) Experimental design to measure the propagation of electrical signals to the same-side systemic leaf after a three-leaflet wound. (b) The amplitude and duration of SWP were measured in both the local (wounded) and systemic (same side) leaf of chickpea. Black and red dots represent the position of electrodes e1 and e2 respectively. The black lines on the leaflet depict the wounded area, with approximately 50% of the leaflet surface wounded using forceps. Recordings were measured for 30 minutes. Data represented are means ± SD. Supplementary Figure 4. Evaluation of DCFDA cell penetration: To control for dye penetration, leaflets were initially treated with 20 µM DCFDA followed by 1 mM H2O2 treatment, and subsequently imaged for ROS fluorescence (upper panel). Leaflets treated with DCFDA alone served as the negative control (lower panel). [file KPSB_A_2567930_SM8672.docx]

Supplementary Figure 1


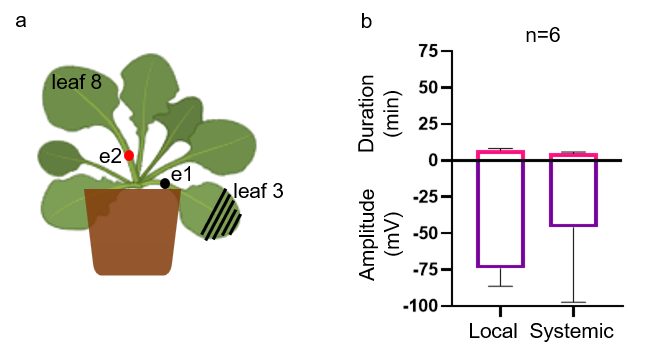


**Supplementary Figure 1.** **SWP pattern in *Arabidopsis*:** SWP was measured in 6-week-old Col-0 lines of *Arabidopsis* using the PhytlSigns instrument. Leaf 3 was wounded, and signals were recorded in both wounded local leaf-3 and systemic leaf-8. **(a)** schematic of the experimental setup for monitoring wound-induced SWP changes, **(b)** Amplitude and duration of SWP in the local and systemic leaf. Data represented are means ± SD.

Supplementary Figure 2


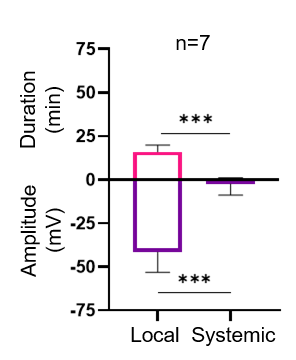


**Supplementary Figure 2. Electrical signaling in seven to nine-day-old chickpea plants:** The electrical signaling was measured in smaller plants with shorter internodal distances. The graph represents amplitude and duration of SWP observed in chickpea seedlings following the wounding of three-terminal leaflets. Data represented are means ± SD. Unpaired, two-tailed Students’ t-test * p<0.05.

Supplementary Figure 3


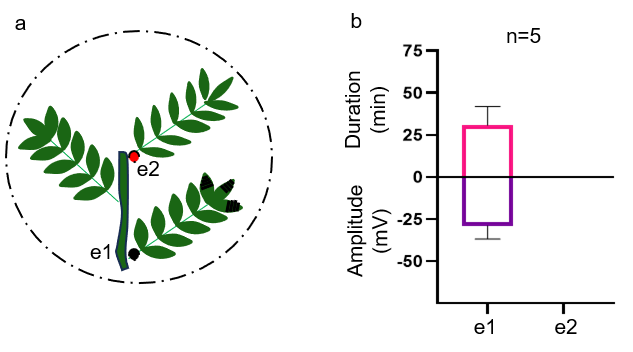


**Supplementary Figure 3. SWP fails to propagate to the same-side systemic leaf:** (**a**) Experimental design to measure the propagation of electrical signals to the same-side systemic leaf after a three-leaflet wound. **(b)** The amplitude and duration of SWP were measured in both the local (wounded) and systemic (same side) leaf of chickpea. Black and red dots represent the position of electrodes e1 and e2 respectively. The black lines on the leaflet depict the wounded area, with approximately 50% of the leaflet surface wounded using forceps. Recordings were measured for 30 minutes. Data represented are means ± SD.

Supplementary Figure 4


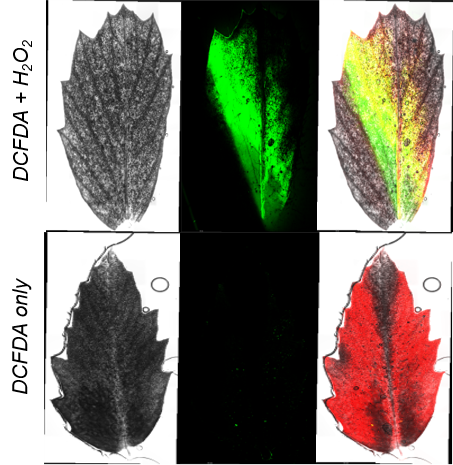


**Supplementary Figure 4. Evaluation of DCFDA cell penetration**: To control for dye penetration, leaflets were initially treated with 20 µM DCFDA followed by 1 mM H_2_O_2_ treatment, and subsequently imaged for ROS fluorescence (upper panel). Leaflets treated with DCFDA alone served as the negative control (lower panel).
